# Impaired Fibrinolytic Potential Predicts Oxygen Requirement in COVID-19

**DOI:** 10.3390/jpm12101711

**Published:** 2022-10-13

**Authors:** Julie Wang, Kay Weng Choy, Hui Yin Lim, Prahlad Ho

**Affiliations:** 1 Northern Health, Epping, Melbourne, VIC 3076, Australia; 2 Department of Medicine, Northern Health, University of Melbourne, Parkville, Melbourne, VIC 3052, Australia

**Keywords:** COVID-19, fibrinolysis, fibrin, overall hemostatic potential

## Abstract

Abnormal coagulation and fibrinolysis contributes to the respiratory distress syndrome in COVID-19. We aimed to explore the association of impaired fibrinolytic potential with disease severity and oxygen requirement in hospitalized patients. Adults admitted to hospital with confirmed COVID-19 infection between 1–31 January 2022 were included, corresponding to the first Omicron outbreak in Melbourne, Victoria. The first citrated plasma sample requested within 24 h of the patient’s presentation was obtained and analyzed by the overall hemostatic potential (OHP) assay, a spectrophotometric assay in which fibrin formation (triggered by small amounts of thrombin (OCP)) and fibrinolysis (by the addition of thrombin and tissue plasminogen activator (OHP and OFP%)) were simultaneously measured. There were 266 patients (median 72 years, 52.9% male), of which 49.6% did not require oxygen therapy. COVID-19 severity and requirement for oxygen was significantly associated with higher OCP, OHP, and lower OFP%. Vaccinated individuals compared with non-vaccinated individuals had significantly lower OHP (16.5 vs. 23.1, *p* = 0.015) and higher OFP (72.0% vs. 65.1%, *p* = 0.005), as well as significantly lower AST, ferritin, LDH, CRP, and D-dimer. A multivariate model containing OHP was constructed with the outcome of oxygen requirement, with c-statistic of 0.85 (95%CI 0.81–0.90). In this pilot study, we show a significant correlation between OHP results and requirement for oxygen supplementation in hospitalized patients during a period dominated by the Omicron variant. The results were incorporated into a multivariate model that predicted for oxygen requirement, with high discriminative ability.

## 1. Introduction

Coagulopathy and endothelial dysfunction are key findings in COVID-19 infection [1] and is thought to be a key factor contributing to clinical deterioration. An autopsy series revealed microthrombi within the pulmonary vasculature of more than 80% of cases [2]. Macrovascular thrombi also occur at a greater rate than in other respiratory infections and high rates are observed even in the presence of thromboprophylaxis [3]. On the other hand, a consumptive coagulopathy is less typically encountered in COVID-19 [4].

Acute respiratory distress syndrome (ARDS) is characterized by abnormal fibrin deposition within the alveolar space and contributes to the respiratory dysfunction seen in this condition [5]. Fibrin deposits have also been found in the lung tissue of COVID-19 patients [6]. In ARDS, the coagulation and fibrinolytic systems are dysregulated, and urokinase activity has been shown to be reduced in the bronchoalveolar fluid of these patients [7]. The fibrinolytic system may be a target for treatment in COVID-19, and markers of hypofibrinolysis may be biomarkers that can predict negative outcome. A phase 2 study in COVID-19 patients with respiratory failure found that tissue plasminogen-activator (tPA) bolus improved oxygenation status compared to controls [8]. Increased plasminogen activator inhibitor-1 (PAI-1) levels have been observed and linked to a negative clinical outcome in COVID-19 [9,10]. Although plasma fibrinolytic activity may not be fully representative of the fibrinolytic activity within the alveolar space, evidence presented thus far suggests a relationship between impaired fibrinolytic activity and negative outcome in COVID-19. Additionally, we have previously demonstrated that in COVID-19 patients, the overall hemostatic assay (OHP), a global coagulation assay evaluating fibrin generation and fibrinolytic potential, predicts a higher rate of ventilatory support and ICU admission [11].

The Omicron variant, on the other hand, has significantly altered the COVID-19 management paradigm, owing to its high infectivity rate. Clinical outcomes have also been milder in Omicron outbreaks [12], which may be in part due to the availability of vaccinations. It is unclear what impacts recent SARS-CoV-2 Omicron variants and vaccination have had on the coagulation and fibrinolysis abnormalities seen in COVID-19. Hence, we designed this study to explore differences in fibrin generation and fibrinolysis between an Omicron-dominant, widely vaccinated population and a previously unvaccinated population during the COVID-19 outbreak in 2020. We specifically correlated the role of OHP with disease severity, specifically oxygen requirement, with the goal of identifying patients who can be safely managed outside of the hospital setting.

## 2. Materials and Methods

The study period occurred between 1 to 31 January 2022 at the Northern Hospital, a tertiary teaching hospital in Melbourne, Australia, and corresponded to the first Omicron (B.1.1.159) outbreak [13] in Australian. Results from 2022 were compared with results obtained from a previous study period from 29 March 2020 to 29 September 2020 at the same institution, corresponding with an outbreak of the B.1.338 [14] subtype. Adult patients (aged ≥18 years) with confirmed SARS-CoV-2 on polymerase chain reaction (PCR) test results were identified using the hospital’s electronic medical records system. Plasma samples were collected and processed in the same way for both study periods, namely—the first citrated sample that was requested within 24 h of the patient’s presentation was obtained and residual plasma following clinical use (within 24–48 h of collection) was double-centrifuged at 2500 G for 10 min, then stored at −80 °C. OHP assays were performed using thawed frozen plasma in October/November 2020 for samples collected in 2020, and May/June 2022 for samples collected in 2022, using the methods described in Section 2.1 below. These results were also compared to results from previously collected healthy controls [15].

Clinical data collected from each patient included other laboratory parameters of the first requested blood test within 24 h of presentation, patient demographics, co-morbidities, vaccination status for SARS-CoV-2, requirement for oxygen or assisted ventilation at any point during the admission, and patient outcomes. Patients were followed up from time of hospital admission to discharge or death, with follow-up censored at time of hospital discharge. Patients were considered fully vaccinated if they have received two or more doses of COVID-19 vaccination. The primary outcome was requirement for any oxygen therapy at any point during the hospital admission. Secondary outcomes were in-hospital clinical deterioration, defined as the requirement for increased ventilatory support (high-flow oxygen, non-invasive and invasive ventilation), admission to intensive care unit, or death attributed to COVID-19 infection. Asymptomatic patients were defined as those patients without symptoms of COVID-19 throughout their hospital admission and were admitted for another indication. Ethics approval was obtained from the Alfred Hospital Ethics Committee (Project ID 67614). As the samples were surplus following clinical investigations, waiver of consent was allowed.

### 2.1. OHP Assay

**Figure 1:**
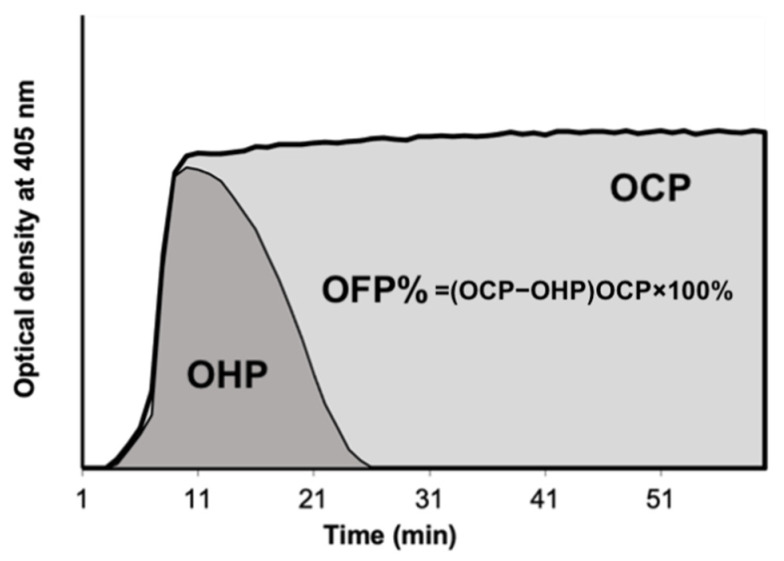
Overall Hemostatic Potential (OHP) assay. OCP is the area under the curve (AUC) of the OD curve obtained by the addition of thrombin (0.006 IU/mL) with CaCl_2_. OHP is the AUC of the curve by adding thrombin (0.006 IU/mL), CaCl_2_ and tPA. The OFP% is the difference between the AUCs OCP and OHP. Abbreviations: OCP overall coagulation potential; OHP overall haemostatic potential; OFP% overall fibrinolytic potential.

The OHP assay is derived from a fibrin aggregation curve created by multiple platelet-poor plasma spectrophotometric measurements (Figure 1). 75 μL of PPP that had been thawed at 37 °C was added to wells containing either (i) Tris, NaCl, and CaCl_2_ at a final concentration of 66 nM Tris, 130 mM NaCl, 35 mM CaCl_2_, pH 7.0), thrombin (0.006 IU/mL), to produce the overall coagulation potential (OCP) or (ii) Tris, NaCl, CaCl_2_, thrombin, and tissue plasminogen activator (tPA) (600 ng/mL) to produce the overall haemostatic potential (OHP). The FLUOstar Optima (BMG Labtech, Ortenberg, Germany) plate reader is used to calculate the two fibrin-aggregation curves (OCP and OHP) cumulatively at 405 nm. The total fibrinolytic potential is determined by the difference between the two curves’ areas underneath them (OFP%). Duplicate tests were run on each sample.

### 2.2. Statistical Analysis

Stata version 17.0 was used to conduct the statistical analysis (StataCorp, College Stations, TX, USA). Continuous variables were presented as means and standard deviation for normally distributed variables, and t-test or ANOVA used to determine differences between groups. For variables with non-normal distribution, results were displayed as medians and interquartile ranges (IQR), and differences between groups determined by the Wilcoxon (rank-sum) test. For multivariate analysis, non-normally distributed variables were transformed to normal distribution prior to linear regression. Categorical variables were shown as counts and frequencies, and differences between groups determined by Chi-squared test. Statistical significance was set at a *p*-value of less than 0.05. 

A multivariate predictive model was constructed with oxygen requirement as the primary endpoint. Univariate logistic regression was first conducted to identify variables associated with oxygen requirement. In the multivariable analysis, variables with *p*-values lower than 0.2 were considered. Missing values were addressed by multiple imputation with chained equations. Candidate final multivariate models were found using backwards stepwise logistic regression. Comparing model fit and choosing the best model involved using C-statistics (area under the receiver operating curve), Schwarz’ Bayesian Information Criterion, Akaike Information Criterion, and the Hosmer-Lemeshow test. Internal validation of the model was performed by bootstrapping with 100 iterations (with each iteration having 20 imputations for missing data).

## 3. Results

During the 2022 study period, 266 COVID-19 positive patients (median age 72 years, interquartile range (IQR) 57–82 years; 52.9% male) were hospitalized and had residual citrated plasma stored. Patients who did not require oxygen during their entire admission comprised 49.6% of the cohort. Table 1 displays results of patients from 2022 and 2020 study periods, as well as normal controls. Patients in the 2022 study period showed significantly higher OCP, OHP, D-dimer and lower OFP compared to normal controls. OHP markers were not significantly different between 2020 and 2022 groups. Patients in 2020 were significantly more likely to be from residential care facilities (30.2% vs. 6.8%, *p* < 0.001), require intensive care unit admission (15.5% vs. 8.3%, *p* = 0.033) and more likely to die (17.4% vs. 6.9%, *p* = 0.002). Many of the patients from residential care facilities were deemed not for aggressive interventions (e.g., assisted ventilation) [16].

Table 2 shows the clinical characteristics, laboratory markers, and OHP assay results for the study patients, organized by disease severity. Age, lower lymphocyte and platelet counts, as well as higher C-reactive protein (CRP), lactate dehydrogenase (LDH), and ferritin levels were all associated with increasing disease severity. After adjusting for age and gender, D-dimer was not significantly different between disease severity groups (*p* = 0.07). Increased disease severity was significantly associated with higher OCP, OHP, and lower OFP%.

Table 3 displays 2022 results according to vaccination status, and compares unvaccinated 2022 patients against 2020 patients, who were all unvaccinated. Although fully vaccinated individuals were less likely to require assisted ventilation and had a lower risk of death (18.4% vs. 4.3%, *p* < 0.001) and clinical deterioration (44.0% vs. 22.7%, *p* = 0.002), rates of oxygen requirement did not differ between vaccinated and unvaccinated groups (56% vs. 49.1%, *p* = 0.38). Fully vaccinated patients, even at presentation, had significantly lower OHP (16.5 vs. 23.1, *p* = 0.015) and higher OFP (72.0% vs. 65.1%, *p* = 0.005), as well as significantly lower AST, ferritin, LDH, CRP, and D-dimer. OCP was not significantly different after adjusting for sex. Compared to patients from the 2020 study period, unvaccinated patients in 2022 showed significantly higher rates of assisted ventilation (38.0% vs. 18.1%, *p* = 0.006) and equivalent rates of death (44.0% vs. 33.6%, *p* = 0.88).

Further analysis was performed according to OHP quartiles. Persons in the highest OHP quartile had an increased risk of requiring oxygen (OR 1.84, 95%CI 1.42–2.37), assisted ventilation (OR 1.65, 95%CI 1.24–2.21) and clinical deterioration (OR 1.41, 95%CI 1.09–1.84), but no difference was seen in death rate (4.7 vs. 8.1%, *p* = 0.44) (Table 4). Of the seven patients in the first quartile of OHP who required ICU admission, three had asymptomatic COVID-19 infection and required ICU level care for other issues (paracetamol overdose, complete heart block and Sheehan’s syndrome). Only 1 (1.5%) venous thromboembolic event occurred in each quartile, and there were no differences with respect to arterial thrombotic events (4 vs. 2 events, *p* = 0.42).

**Figure 2:**
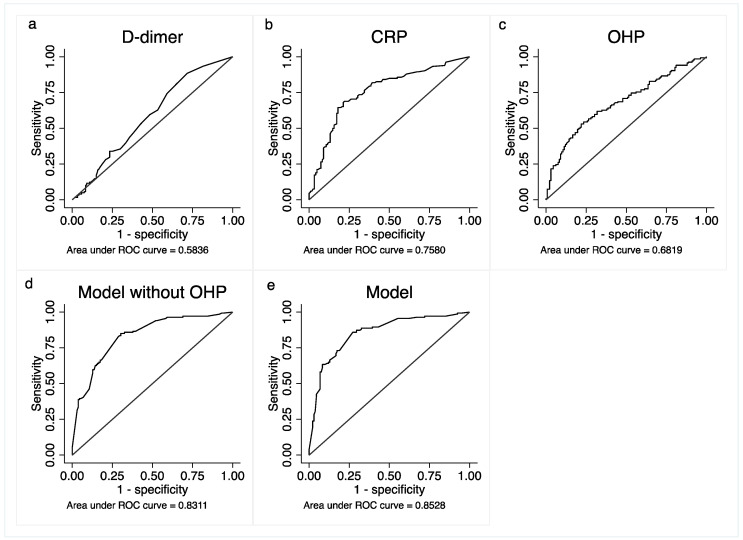
Receiver operating curves with outcome being oxygen requirement. (**a**) D-dimer (**b**) CRP; (**c**) OHP (**d**) multivariate model without OHP (**e**) multivariate model with OHP.

A multivariate model was constructed for the primary outcome of requirement for oxygen (Table 5). The resultant model contained the variables age, LDH, CRP, platelet count, COPD and OHP, with c-statistic of 0.853 (95%CI 0.81–0.90). Removing OHP from the model led to a decrease in the c-statistic (Figure 2). The model was internally validated by bootstrapping with 100 iterations, with a resultant c-statistic of 0.855 (0.80–0.91). The area under the curve of OHP as a predictor for oxygen requirement was higher than D-dimer (0.68 vs. 0.58).

## 4. Discussion

In this study of 266 hospitalized COVID-19 patients from January 2022, we show that OHP assay results were significantly correlated with oxygen requirement and severity of COVID-19 infection, with increasing fibrin generation (OCP and OHP) and decreased fibrinolysis (OFP) corresponding to worsening disease severity. Patients in the top quartile of OHP results, which reflects both fibrin formation as well as lysis, were more likely to require oxygen therapy (OR: 1.84 (95%CI 1.42–2.37)) and assisted ventilation (OR: 1.65 (95% CI 1.24–2.21)). This is despite a lack of relationship between OHP and venous or arterial thrombosis, which may in part be due to widespread use of thromboprophylaxis in COVID-19 patients. 

These findings support the assertion that dysregulation of the coagulation and fibrinolysis system plays an integral part in the abnormal changes seen in the lungs of patients with COVID-19 [16,17]. In severe COVID-19, a process of immunothrombosis results from cytokine storm, endothelial dysfunction and hypofibrinolysis [18], resulting in pulmonary microthrombi and abnormal turnover of fibrin [19]. These changes may be directly activated by the SARS-CoV-2 virus causing downregulation of ACE-2 expression in lung tissue resulting in dysregulation of the renin-angiotensin system leading to vasoconstriction, endothelial activation and disturbing of the natural anticoagulant systems [20]. Inflammation also leads to increased synthesis of fibrinogen in the liver [21] and lung epithelium [22], further compounding pulmonary fibrin deposition. CRP is also increased in inflammation, which promotes PAI-1 release from endothelial cells [23]. Abnormal amounts of fibrinolytic markers have been associated with poor outcome in COVID-19, including PAI-1 [10,24], tPA [9], TAFI [25] and plasminogen [26]. These impairments in the fibrinolytic pathway have also been detected by viscoelastic testing [17,24] and OHP assay [11], although a direct comparison between the different methods has not yet been undertaken.

According to epidemiological studies, the Omicron variants are less severe than previous variants [27,28]. Our local data supports this [29], with a lower proportion of patients requiring hospitalization in 2022 and fewer serious outcomes compared to previous outbreaks. The improved outcomes were also likely to have resulted from widespread vaccination by the time of the 2022 study period, with 81.2% having received two or more doses in our study cohort. Interestingly, patients in 2022 with fewer than two doses of vaccination had higher rates of assisted ventilation compared to patients in 2020 (38.0% vs. 18.1%, *p* = 0.003) and equivalent rates of death (18.4% vs. 17.4%, *p* = 0.001), indicating that hospitalized unvaccinated persons infected with the Omicron variant still have a similar risk of negative outcome compared to previous variants. Another interesting finding from our data was that markers like CRP, AST and ferritin, as well as OHP, were significantly higher in non-fully vaccinated people, even on presentation to the hospital. Our findings highlight the importance of vaccination, even in a less severe variant such as Omicron.

Despite the availability of vaccinations and potentially less severe COVID-19 variants in recent outbreaks, COVID-19 remains intensive in its utilization of healthcare resource, particularly due to its increased infectivity, ability to escape antibodies from previous vaccination or infection, as well as the concurrent resurgence of other viruses such as influenza. Given the large number of COVID-19 infections during the Omicron era, it is imperative to identify patients who can be safely managed at home. OHP is a unique marker of fibrinolysis, which is associated with poor ventilation outcomes, and from our work shown to be superior to D-dimer in predicting oxygen requirement (c-stat 0.68 vs. 0.58). Hence, we refined our previously published multivariate model [29] to include OHP. The resulting model, confirmed with internal validation by bootstrapping, had an excellent c-statistic of 0.85 and is easy to use with widely available tests. This model may identify patients who may be managed in the outpatient setting or in subacute facilities, reducing the demand on healthcare resources. External validation is required however, before this model can be used in clinical decision making.

We acknowledge that our study has several limitations. Plasma samples were not collected specifically for OHP, but rather on the presence of residual plasma collected as part of routine clinical work. This could have resulted in subject selection bias, as well as non-standardized specimen collection and handling. Some patients were excluded if citrated plasma was not available and hence a complete consecutive assessment may not be possible. Clinical data were gathered retrospectively and were subject to the documentation quality and completeness in medical records. Normal control subjects were significantly younger than patient cohorts from 2020 and 2022 (Table 1). This could have affected comparisons for OHP results as age is known to affect fibrin generation and fibrinolytic potentials [30]. However, we have attempted to account for this by performing adjustment for age using multivariate linear regression. Routine testing to confirm the variant subtype was not performed at our institution during either collection period. Despite the lack of local variant data, it is worth noting that the Omicron variant was first detected in Victoria, Australia in mid-December 2021, and by January 2022 comprised at least 80% of positive COVID samples at the Victorian state reference laboratory [14], implying that the Omicron variant infected the majority of patients during the 2022 study period. Criticisms have also been levelled at COVID-19 risk prediction models [31], which were found to be at risk of bias, and should be ideally prospectively validated in external settings different to the conditions of the derivation study. The results of our study are hypothesis generating and pilot in nature and require confirmation in larger and external test settings.

## 5. Conclusions

The results of our pilot study appear to demonstrate a significant correlation between the overall hemostatic assay, a fibrin generation assay, and the severity of COVID-19 infection, defined by the requirement for supplemental oxygen. A multivariate model incorporating OHP was developed that could be easily applied to identify patients at low risk of requiring oxygen and thus be managed safely at home. Further validation in a large cohort is required.

## Figures and Tables

**Table 1:** Baseline characteristics, laboratory results and clinical outcomes of normal controls compared with COVID-19 patients from 2020 and 2022. Data are *n* (%), and median (interquartile range, IQR), unless specified otherwise.

	Normal Controls (NC)	2020	2022	*p*-Value NC vs. 2022	*p*-Value 2020 vs. 2022
*n*	153	116	266		
Age (years)	39.0 (24.0, 57.0)	67.0 (50.0, 84.0)	72.0 (57.0, 82.0)	**<0.001**	0.38
Male	55 (35.9%)	57 (49.1%)	138 (51.9%)	**0.002**	0.62
Fully vaccinated (>=2 doses)	0 (0.0%)	0 (0.0%)	216 (81.2%)	**<0.001**	**<0.001**
From residential care facility	0 (0.0%)	35 (30.2%)	18 (6.8%)	**0.001**	**<0.001**
Hypertension		67 (57.8%)	131 (49.2%)		0.13
Diabetes		39 (33.6%)	100 (37.6%)		0.46
COPD		6 (5.2%)	23 (8.6%)		0.24
IHD		21 (18.1%)	56 (21.1%)		0.51
CKD		13 (11.2%)	46 (17.3%)		0.13
CCF		16 (13.8%)	34 (12.8%)		0.79
Malignancy		2 (1.7%)	23 (8.6%)		**0.012**
Weight (kg)		77.0 (62.0, 96.0)	77.0 (67.0, 93.9)		0.54
Venous thrombosis		2 (1.7%)	4 (1.5%)		0.87
Intensive care unit		18 (15.5%)	22 (8.3%)		**0.033**
Assisted ventilation		21 (18.1%)	59 (22.2%)		0.37
Death from COVID-19		20 (17.4%)	18 (6.9%)		**0.002**
Haemoglobin (g/L), mean (SD)	144.9 (12.5)	132.2 (19.4)	132.4 (19.9)	<0.001 *	0.96
Neutrophils (×10^9^/L)	3.2 (2.5, 4.1)	4.3 (2.8, 6.8)	4.4 (3.2, 6.6)	<0.001 *	0.91
Lymphocytes (×10^9^/L)		0.9 (0.7, 1.3)	1.0 (0.7, 1.4)		0.57
Neutrophil/Lymphocyte ratio		4.3 (2.5, 7.5)	4.2 (2.9, 7.7)		0.89
Platelets (×10^9^/L)	243.0 (215.0, 283.0)	222.5 (168.5, 275.5)	211.0 (157.0, 264.0)	0.16	0.25
Creatinine (µmol/L),		77.0 (58.0, 99.0)	85.0 (63.0, 115.0)		**0.032**
AST (unitls/L)		29.0 (22.0, 44.0)	40.0 (27.0, 54.0)		**<0.001**
Bilirubin (µmol/L)		8.0 (5.0, 11.0)	9.0 (7.0, 13.0)		**0.012**
Ferritin (ug/L)		411.5 (181.5, 950.0)	343.0 (134.0, 777.0)		0.10
LDH (units/L)		395.0 (179.0, 919.0)	287.0 (215.0, 397.0)		**0.012**
CRP (mg/L)		47.0 (15.5, 90.0)	42.0 (9.0, 97.0)		0.51
D-dimer (mg/L FEU)	0.2 (0.1, 0.3)	0.7 (0.4, 1.4)	0.9 (0.5, 1.7)	<0.001 *	**0.026**
OCP (units)	34.5 (29.0, 43.3)	59.8 (44.7, 74.6)	58.1 (44.9, 81.0)	<0.001 *	0.65
OHP (units)	6.4 (4.8, 9.5)	16.4 (12.1, 24.3)	17.3 (11.1, 27.1)	<0.001 *	0.69
OFP (%)	81.1 (77.5, 84.1)	70.2 (63.5, 75.7)	71.5 (63.6, 77.3)	<0.001 *	0.59

Abbreviations: COPD, chronic obstructive pulmonary disease; IHD, ischemic heart disease; CKD, chronic kidney disease; CCF, congestive cardiac failure; AST, aspartate transaminase; LDH, lactate dehydrogenase; CRP, C-reactive protein; OCP, overall coagulation potential; OHP, overall hemostatic potential; OFP, overall fibrinolytic potential. * *p*-values adjusted for age and sex; boldened values signify *p* < 0.05.

**Table 2:** Baseline characteristics, laboratory results and clinical outcomes of COVID-19 patients from 2022 categorized by disease severity. Data are *n* (%), and median (interquartile range, IQR), unless specified otherwise.

	Asymptomatic	Symptomatic but Did Not Require Oxygen Therapy	Required Oxygen Therapy but Did Not Deteriorate	Clinically Deteriorated	*p*-Value
*n*	41	86	68	71	
Age (years)	70.0 (40.0, 81.0)	71.0 (42.0, 83.0)	73.0 (67.0, 79.5)	73.0 (62.0, 83.0)	**0.011**
Male	21 (51.2%)	34 (39.5%)	41 (60.3%)	42 (59.2%)	**0.034**
Fully vaccinated (>=2 doses)	34 (82.9%)	73 (84.9%)	60 (88.2%)	49 (69.0%)	**0.019**
Days from symptom onset to admission	3.0 (1.0, 9.0)	3.0 (1.0, 6.0)	6.5 (3.0, 9.0)	4.0 (2.0, 8.0)	0.19 *
Hypertension	16 (39.0%)	34 (39.5%)	43 (63.2%)	38 (53.5%)	**0.013**
Diabetes	11 (26.8%)	26 (30.2%)	32 (47.1%)	31 (43.7%)	0.052
COPD	2 (4.9%)	3 (3.5%)	8 (11.8%)	10 (14.1%)	0.068
IHD	5 (12.2%)	14 (16.3%)	19 (27.9%)	18 (25.4%)	0.12
CKD	2 (4.9%)	15 (17.4%)	12 (17.6%)	17 (23.9%)	0.085
CCF	3 (7.3%)	13 (15.1%)	7 (10.3%)	11 (15.5%)	0.50
Malignancy	4 (9.8%)	6 (7.0%)	4 (5.9%)	9 (12.7%)	0.48
Smoking history					0.093
Non-smoker	21 (61.8%)	54 (72.0%)	32 (52.5%)	35 (54.7%)	
Smoker	5 (14.7%)	6 (8.0%)	9 (14.8%)	4 (6.2%)	
Ex-smoker	8 (23.5%)	15 (20.0%)	20 (32.8%)	25 (39.1%)	
Weight (kg)	70.0 (63.0, 86.0)	75.0 (66.8, 89.0)	79.0 (74.0, 95.8)	79.5 (67.0, 103.0)	0.004 *
Venous thrombosis	0 (0.0%)	2 (2.3%)	1 (1.5%)	1 (1.4%)	0.80
Arterial thrombosis	2 (4.9%)	2 (2.3%)	8 (11.8%)	3 (4.2%)	0.076
Intensive care unit	0 (0.0%)	0 (0.0%)	0 (0.0%)	23 (32.4%)	**<0.001**
Assisted ventilation	0 (0.0%)	0 (0.0%)	0 (0.0%)	59 (83.1%)	**<0.001**
Death caused by COVID-19	0 (0.0%)	0 (0.0%)	0 (0.0%)	18 (27.7%)	**<0.001**
Haemoglobin (g/L), mean (SD)	128.4 (24.7)	130.2 (18.6)	137.7 (16.5)	132.2 (20.5)	0.07 *
Neutrophils (×10^9^/L)	4.5 (2.9, 6.7)	4.2 (3.2, 6.3)	4.3 (3.3, 6.3)	4.8 (3.2, 6.8)	0.25 *
Lymphocytes (×10^9^/L)	1.3 (0.9, 1.9)	1.0 (0.7, 1.5)	0.9 (0.6, 1.2)	0.9 (0.6, 1.1)	0.001 *
Neutrophil/Lymphocyte ratio	4.2 (2.3, 6.8)	4.2 (3.1, 8.2)	4.2 (2.9, 6.8)	4.6 (2.9, 7.7)	0.47 *
Platelets (×10^9^/L)	247.0 (166.0, 305.0)	218.5 (163.0, 275.0)	199.5 (158.5, 234.5)	190.0 (141.0, 243.0)	0.007 *
Creatinine (µmol/L),	71.0 (58.0, 97.0)	84.5 (62.0, 115.5)	93.0 (67.0, 114.0)	92.0 (67.0, 135.0)	0.32 *
AST (units/L)	31.0 (24.0, 48.0)	34.0 (26.0, 48.0)	41.0 (29.0, 57.0)	43.0 (32.0, 65.0)	<0.001 *
Bilirubin (µmol/L)	10.5 (7.0, 13.0)	9.0 (7.0, 13.0)	9.0 (7.0, 14.0)	9.0 (6.0, 12.0)	0.19 *
Ferritin (ug/L)	168.0 (77.0, 316.0)	242.0 (79.5, 468.0)	510.0 (243.0, 905.0)	483.5 (259.5, 1098.0)	<0.001 *
LDH (units/L)	220.0 (187.0, 310.0)	247.5 (210.0, 327.5)	304.0 (227.0, 376.0)	397.5 (284.0, 502.0)	<0.001 *
CRP (mg/L)	9.5 (2.5, 29.0)	17.0 (6.0, 45.0)	64.0 (19.0, 104.5)	86.0 (39.0, 130.0)	<0.001 *
D-dimer (mg/L FEU)	0.8 (0.4, 1.4)	0.8 (0.4, 1.4)	0.9 (0.6, 1.7)	1.2 (0.7, 2.2)	0.07 *
OCP (units)	50.9 (43.0, 59.5)	53.0 (43.2, 70.1)	69.7 (53.3, 91.7)	70.8 (45.0, 84.1)	0.001 *
OHP (units)	13.2 (9.3, 18.5)	15.0 (10.3, 23.2)	19.8 (13.3, 30.8)	22.7 (12.9, 35.4)	<0.001 *
OFP (%)	74.0 (69.5, 78.6)	72.4 (65.3, 78.3)	71.8 (62.5, 76.5)	67.3 (56.0, 74.5)	<0.001 *

Abbreviations: COPD, chronic obstructive pulmonary disease; IHD, ischemic heart disease; CKD, chronic kidney disease; CCF, congestive cardiac failure; AST, aspartate transaminase; LDH, lactate dehydrogenase; CRP, C-reactive protein; OCP, overall coagulation potential; OHP, overall hemostatic potential; OFP, overall fibrinolytic potential. * *p*-values adjusted for sex and age; boldened values signify *p* < 0.05.

**Table 3:** Baseline characteristics, laboratory results and clinical outcomes of COVID-19 patients from 2022 categorized by vaccination status. Data are *n* (%), and median (interquartile range, IQR), unless specified otherwise.

	2020	2022 Unvaccinated (<2 Doses)	2022 Vaccinated (≥2 Doses)	*p*-Value 2022 Unvaccinated vs. Vaccinated	*p*-Value 2020 vs. 2022 Unvaccinated
*n*	116	50	216		
Age (years)	67.0 (50.0, 84.0)	72.0 (56.0, 81.0)	72.0 (57.5, 82.0)	0.98 *	0.66
Male	57 (49.1%)	21 (42.0%)	117 (54.2%)	0.12	0.40
Weight (kg)	77.0 (62.0, 96.0)	80.0 (63.0, 100.0)	77.0 (67.0, 92.0)	0.31 *	0.39
Fully vaccinated	0 (0.0%)	0 (0.0%)	216 (100%)	**<0.001**	1.00
Days from symptom onsent to admission	Not collected	3.0 (1.0, 7.0)	4.0 (2.0, 8.0)	0.63	N/A
Venous thrombosis	2 (1.7%)	0 (0.0%)	4 (1.9%)	0.33	0.35
ICU	18 (15.5%)	7 (14.0%)	16 (7.4%)	0.14	0.80
Assisted ventilation	21 (18.1%)	19 (38.0%)	40 (18.5%)	**0.003**	**0.006**
Deterioration	39 (33.6%)	22 (44.0%)	49 (22.7%)	**0.002**	0.20
Death from COVID	20 (17.4%)	9 (18.4%)	9 (4.3%)	**<0.001**	0.88
Haemoglobin (g/L), mean (SD)	132.2 (19.4)	132.4 (15.3)	132.3 (20.8)	0.91 *	0.95
Neutrophils (×10^9^/L)	4.3 (2.8, 6.8)	4.2 (3.3, 7.6)	4.5 (3.2, 6.4)	0.29 *	0.47
Lymphocytes (×10^9^/L)	0.9 (0.7, 1.3)	0.9 (0.7, 1.3)	1.0 (0.7, 1.5)	0.27 *	0.60
Neutrophil/Lymphocyte ratio	4.3 (2.5, 7.5)	4.4 (3.3, 8.0)	4.2 (2.6, 7.6)	0.21 *	0.36
Platelets (×10^9^/L)	222.5 (168.5, 275.5)	199.0 (163.0, 282.0)	214.0 (157.0, 263.5)	0.96 *	0.33
Creatinine (µmol/L),	77.0 (58.0, 99.0)	78.0 (60.0, 108.0)	86.5 (64.0, 115.0)	0.43 *	0.53
AST (units/L)	29.0 (22.0, 44.0)	42.0 (31.0, 65.0)	38.0 (27.0, 53.0)	0.048 *	**<0.001**
Bilirubin (µmol/L)	8.0 (5.0, 11.0)	9.0 (6.0, 12.0)	9.0 (7.0, 13.5)	0.60 *	0.093
Ferritin (ug/L)	411.5 (181.5, 950.0)	866.0 (264.0, 1415.0)	306.0 (130.0, 596.5)	<0.001 *	0.13
LDH (units/L)	395.0 (179.0, 919.0)	320.0 (234.0, 471.0)	278.0 (215.0, 361.0)	0.013 *	0.48
CRP (mg/L)	47.0 (15.5, 90.0)	63.5 (11.0, 120.0)	32.5 (9.0, 88.5)	0.050 *	0.29
D-dimer (mg/L FEU)	0.7 (0.4, 1.4)	1.3 (0.7, 1.9)	0.8 (0.5, 1.7)	0.012 *	**<0.001**
OCP (units)	59.8 (44.7, 74.6)	67.6 (47.9, 80.1)	56.6 (44.2, 81.1)	0.20 *	0.21
OHP (units)	16.4 (12.1, 24.3)	23.1 (14.1, 34.9)	16.5 (10.7, 24.7)	0.015 *	**0.014**
OFP (%)	70.2 (63.5, 75.7)	65.1 (54.5, 76.4)	72.0 (65.4, 77.4)	0.005 *	0.083

Abbreviations: AST, aspartate transaminase; LDH, lactate dehydrogenase; CRP, C-reactive protein; OCP, overall coagulation potential; OHP, overall hemostatic potential; OFP, overall fibrinolytic potential. * *p*-values adjusted for sex; boldened values signify *p* < 0.05.

**Table 4:** Clinical outcome of COVID-19 patients from 2022, comparing those with the highest OHP quartile to the lowest OHP quartile. Data are *n* (%), and median (interquartile range, IQR), unless specified otherwise.

	OHP Quartile 1	OHP Quartile 4	*p*-Value
*n*	67	65	
Age (years)	73.0 (47.0, 84.0)	72.0 (61.0, 79.0)	0.61 *
Male	30 (44.8%)	37 (56.9%)	0.16
D-dimer (mg/L FEU)	2.28	2.94	0.001 *
Fully vaccinated	59 (88.1%)	43 (66.2%)	**0.003**
Days from symptom onset to admission	3.0 (1.0, 6.0)	7.0 (3.0, 11.0)	**<0.001**
Weight (kg)	74.5 (62.2, 87.5)	78.2 (68.0, 100.0)	0.032 *
Venous thrombosis	1 (1.5%)	1 (1.5%)	0.98
Arterial thrombosis	4 (6.0%)	2 (3.1%)	0.42
Oxygen requiring	23 (34.3%)	50 (76.9%)	**<0.001** **(OR: 1.84 (95%CI 1.42–2.37))**
Intensive care unit	7 (10.4%)	9 (13.8%)	0.55
Inotropes	5 (7.5%)	1 (1.6%)	0.11
Assisted ventilation	9 (13.4%)	27 (41.5%)	**<0.001** **(OR: 1.65 (95%CI 1.24–2.21))**
Deterioration	13 (19.4%)	27 (41.5%)	**0.006** **(OR: 1.41 (95%CI 1.09–1.84))**
Death from COVID-19	3 (4.7%)	5 (8.1%)	0.44

Abbreviations: OHP, overall hemostatic potential; OR, odds ratio * *p*-values adjusted for sex; boldened values signify *p* < 0.05.

**Table 5:** Multivariate logistic model for prediction of oxygen requirement in COVID-19 patients from 2022.

O_2_	Odds Ratio	Coefficient	Standard Error of Odds Ratio	t-Score	*p*-Value	95% Confidence Interval of Odds Ratio	
Age ≥ 50 (years)	4.60	1.53	2.13	3.30	**0.001**	1.86	11.38
LDH > 250 (units/L)	2.28	0.83	0.80	2.37	**0.018**	1.15	4.52
CRP > 50 (mg/L)	4.27	1.45	1.50	4.11	**<0.001**	2.14	8.52
Plt ≥ 250 (×10^9^/L)	0.23	−1.47	0.09	−3.89	**<0.001**	0.11	0.48
OHP > 20 (units)	3.23	1.17	1.24	3.05	**0.002**	1.52	6.86
COPD	2.70	0.99	1.56	1.72	0.085	0.87	8.35

Abbreviations: COPD, chronic obstructive pulmonary disease; IHD, ischemic heart disease; LDH, lactate dehydrogenase; CRP, C-reactive protein; boldened values signify *p* < 0.05.

## Data Availability

Not applicable.
